# Development of an Electrostatic Comb-Driven MEMS Scanning Mirror for Two-Dimensional Raster Scanning

**DOI:** 10.3390/mi12040378

**Published:** 2021-04-01

**Authors:** Qiang Wang, Weimin Wang, Xuye Zhuang, Chongxi Zhou, Bin Fan

**Affiliations:** 1 State Key Laboratory of Optical Technologies on Nano-Fabrication and Micro-Engineering, Institute of Optics and Electronics, Chinese Academy of Sciences, Chengdu 610209, China; battery007@163.com (Q.W.); cxzhou@ioe.ac.cn (C.Z.); 2 University of Chinese Academy of Sciences, Beijing 100049, China; 3 Key Laboratory of Optoelectronic Technology and Systems, Ministry of Education & Defense Key Disciplines Laboratory of Novel Micro-Nano Devices and System Technology, Chongqing University, Chongqing 400044, China; wwm@cqu.edu.cn; 4 Guangxi Key Laboratory of Automatic Detecting Technology and Instruments, Guilin University of Electronic Technology, Guilin 541004, China; 5 School of Mechanical Engineering, Shandong University of Technology, Zibo 255000, China; zxye@sdut.edu.cn

**Keywords:** two-dimensional raster scanning, electrostatic comb-drive actuator, vacuum operation, mechanical coupling, residual stress, in-phase, out-of-phase, parametric resonance

## Abstract

Microelectromechanical System (MEMS)-based scanning mirrors are important optical devices that have been employed in many fields as a low-cost and miniaturized solution. In recent years, the rapid development of Light Detection and Ranging (LiDAR) has led to opportunities and challenges for MEMS scanners. In this work, we propose a 2D electrostatically actuated micro raster scanner with relatively large aperture. The 2D scanner combines a resonant scanning axis driven by an in-plane comb and a quasistatic scanning axis driven by a vertical comb, which is achieved by raising the moving comb finger above the fixed comb finger through the residual stress gradient. The analytic formula for the resonant axis frequency, based on the mechanical coupling of two oscillation modes, is derived and compared with finite element simulation. A prototype is designed, fabricated, and tested, and an overall optical Field-of-View (FoV) of about 60° × 4° is achieved. Finally, some possibilities for further improvement or optimization are discussed.

## 1. Introduction

Microelectromechanical System (MEMS) scanning mirrors have potential applications in miniaturized Light Detection and Ranging (LiDAR) [1,2], projection display [3], and optical coherence tomography (OCT) [4,5], among others. Electrostatic actuator-based micro scanners are one of the most important devices among these, due to their low power consumption, fast response, and integrated circuit (IC) process compatibility [6].

According to the scanning axis, scanners can be divided into one-dimensional (1D) and two-dimensional (2D) scanners [7]. According to the types of vibration, scanners can be divided into resonant and non-resonant (forced vibration, also known as quasistatic) scanning type [8,9]. Thus, 2D scanners have three configurations: double axis with resonant scanning, double axis with non-resonant scanning, and one resonant axis plus one non-resonant axis. Double resonant scanning generates Lissajous scanning patterns [10,11], which have the drawback of higher required resonant frequency than the other two configurations when in the same spatial resolution. Hofmann et al. proposed a 0.8 mm scanner with resonant angle of ±21.5° [12]. Scanners of one resonant and one non-resonant scanning type avoid this shortcoming and can result in raster scanning patterns [13]. In addition, their resonant axis has a larger scanning angle, compared to double non-resonant scanning. In [14], a high performance scanner with this configuration was presented. Double non-resonant scanners are more suitable for vector display and beam steering [15]. Mirrorcle Technologies, Inc. have developed a number of related products [16,17,18].

In this paper, we propose a 4 mm aperture raster scanner, for which the design and experimental measurement are demonstrated. The remaining sections of this paper are organized as follows: In Section 2, the structural design and simulation are presented and analyzed. In Section 3, the fabrication process is described. The experimental testing is detailed in Section 4, and summaries are given in Section 5.

## 2. Design and Simulation

### 2.1. Slow Axis

**Figure 1:**
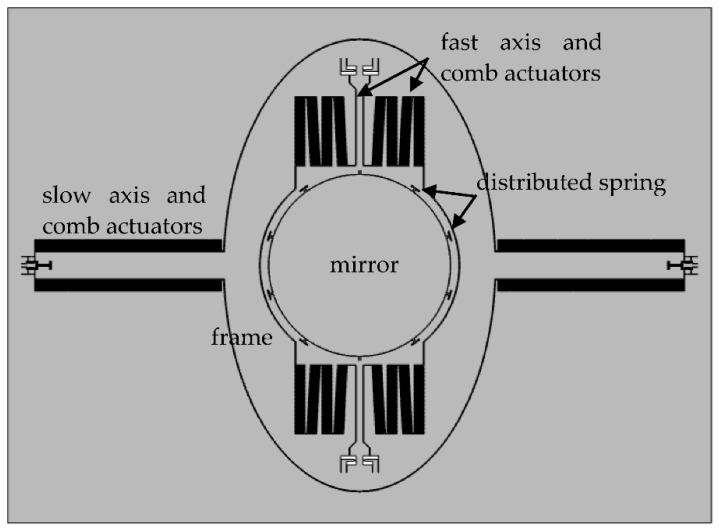
Layout of the proposed scanner.

The scanner utilizes a gimbaled frame, including an inner resonant axis (fast axis) and an outer non-resonant axis (slow axis), as shown in Figure 1. Considering the processing capability, a distributed spring to minimize the dynamic deformation is used [19].

The mirror aperture is 4 mm, which is a little larger than most electrostatic devices, leading to difficulty in improving other parameters. This is because the larger aperture results in greater mass and dynamic deformation and, therefore, requires a greater spring constant and driving force. Electromagnetic and electrothermal actuators are more suitable for this situation, due to their greater force and displacement than electrostatic devices.

**Figure 2:**
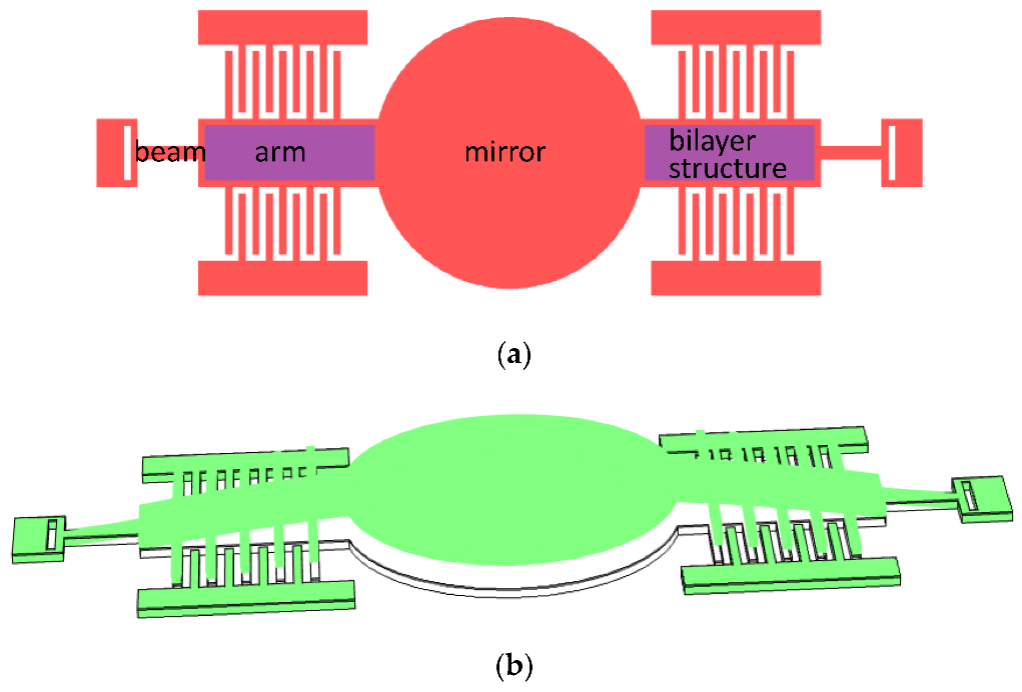
Schematic diagram of the slow axis: (**a**) Top view; and (**b**) stress-induced bending of the mirror, arm, and beam.

To realize the static rotation of the quasistatic axis, vertical comb-drive actuators are utilized. Compared with various methods in the literature [8,20,21], curvature induced by residual stress is employed to form vertical comb pairs, for ease of machining. To demonstrate the formation principle and the lifted moving comb finger of the vertical comb actuator clearly, a simplified schematic diagram of the slow axis is depicted in Figure 2a. Two arms of the slow axis are deposited by a film with large residual compressive stress and a vertical stress gradient is produced by mismatch in the stress between the bilayer structure, resulting in the convex shape of the slow axis, as shown in Figure 2b. The moving comb of the slow axis will be lifted up and the vertical height from the moving comb to the fixed comb increases gradually along the slow axis, similar to the pre-stress comb-drive actuator [22,23].

The curved arms are not anchored directly but, instead, connected to the anchor by a thin beam, which has a much smaller bending stiffness than the arm. Hence, the connection point can be regarded as a simply supported boundary condition, making the moving comb higher.

In order to keep the surface of the mirror smooth, the compressive stress film on the surface of the mirror is etched and removed.

### 2.2. Fast Axis

Due to the use of a gimbaled frame, there are two resonant modes of interest in the fast axis; namely, in-phase (IP) and out-of-phase (OP) modes [24]. Both of these modes arise from the mechanical coupling between two torsion motions: one is the torsion of the mirror around the fast axis, while the other is the torsion of the frame around the fast axis. IP and OP represent the mirror and the frame moving in phase and out of phase, respectively. The relationship between these modes is as follows:

(1) $$\omega_{OP}^{2}=\frac{k_{i}}{2J_{o}}+\frac{\omega_{o}^{2}}{2}+\frac{\omega_{i}^{2}}{2}+\Delta ,$$

(2) $$\omega_{IP}^{2}=\frac{k_{i}}{2J_{o}}+\frac{\omega_{o}^{2}}{2}+\frac{\omega_{i}^{2}}{2}-\Delta ,$$

where

(3) $$\Delta =\frac{\sqrt{-4k_{i}k_{o}J_{i}J_{o}+{\left(J_{i}k_{i}+J_{i}k_{o}+J_{o}k_{i}\right)}^{2}}}{2J_{o}J_{i}},$$

(4) $$\omega_{o}=\sqrt{\frac{k_{o}}{J_{o}}},$$

(5) $$\omega_{i}=\sqrt{\frac{k_{i}}{J_{i}}};$$

*ω_op_* and *ω_ip_* are the resonant angular frequencies of the OP and IP modes, *ω_i_* and *ω_o_* are the resonant frequencies of the two torsion motions, and *k_i_*, *k_o_*, *J_i_*, and *J_o_* are the spring constants and mass moments of inertia of the two torsion motions, respectively.

The calculation formulae of *k_i_*, *k_o_*, *J_i_*, and *J_o_* can be found in [25], but the boundary conditions of *k_o_* and *J_o_* here differ from those in [25]. Therefore, they are derived again in Appendix A (see Equations (A13) and (A18)). Based on the formulas and structural parameters, the calculated frequencies are presented in Table 1. Furthermore, the resonant frequencies and modes were simulated by the COMSOL Multiphysics finite element analysis (FEA) software [26], as listed in Table 1. According to the comparison of the data, the error of the theoretical formula is about 10%.

**Figure 3:**
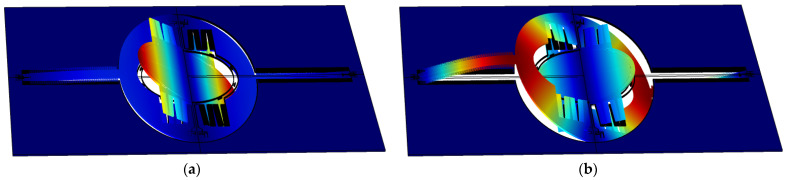
Simulation of resonance modes of the fast axis: (**a**) In-phase; and (**b**) Out-of-phase.

Figure 3 shows the simulated resonant modes, in which the in-phase and out-of-phase modes can be clearly observed. In IP mode, the angles of the mirror and frame have the same sign, while in OP mode the signs are opposite.

## 3. Fabrication

**Figure 4:**
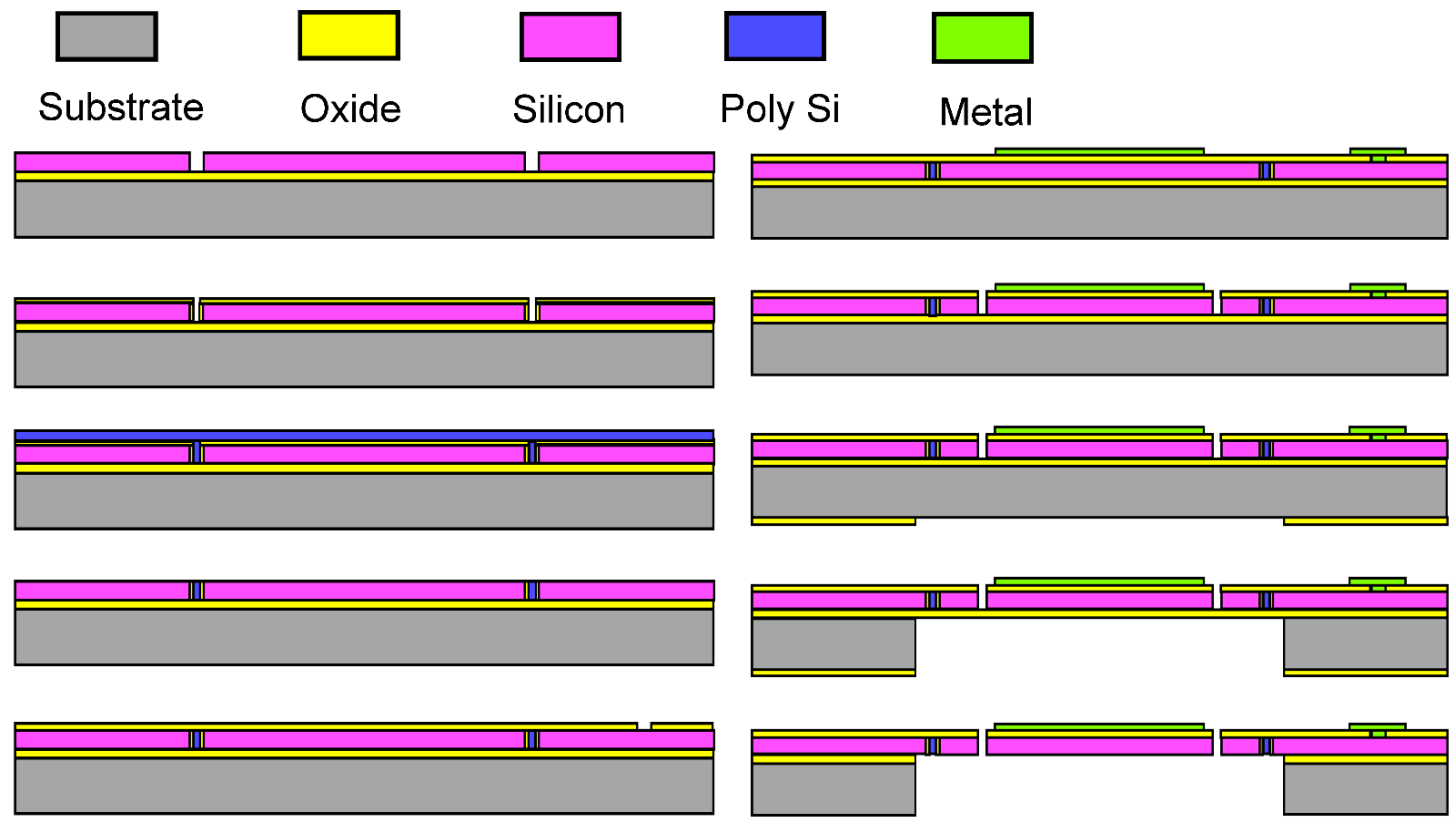
The proposed mirror fabrication process.

The fabrication process and parameters are shown in Figure 4 and Table 2, respectively. A SOI wafer is used as base material. First of all, the wafer is cleaned by buffered hydrofluoric acid (BHF) solution, in order to remove the native oxide layer. Then, isolation trenches are vertically etched and refilled with thermal oxide and polysilicon. After refilling, chemical mechanical polishing (CMP) and dry etching are adopted successively to remove the polysilicon and oxide outside the trench. Next, another silicon dioxide layer is deposited, exposed, and etched, which provides residual stress to curl up the gimbaled frame and insulates the silicon from the metal on it. After that, a metal layer is deposited and patterned. Its functions contain the reflective coating and wires for driving signals. Then, the single crystal silicon is etched by Inductively Coupled Plasma (ICP) to form the device structure, such as comb actuators and beams. Finally, the bottom side of the wafer (i.e., the substrate) is deposited with silicon dioxide by plasma enhanced chemical vapor deposition (PECVD). This layer serves as photoresist mask for substrate etching, and the SiO_2_, substrate and buried oxide layer are patterned step-by-step. Finally, the deposited SiO_2_ is removed.

## 4. Experimental Testing

### 4.1. Profile Testing

**Figure 5:**
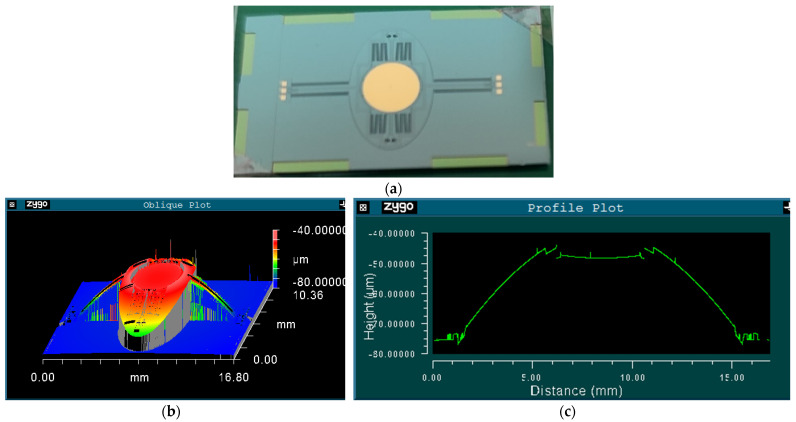
(**a**) Photo of the fabricated prototype; (**b**) Surface profile of the prototype; and (**c**) Cross-sectional view of the prototype along the slow axis.

A photo of the fabricated prototype is shown in Figure 5a. First of all, the surface profile of the scanner was measured by a 3D optical profiling system (Zygo NewView 7300, Zygo Co., Middlefield, OH, USA). As shown in Figure 5b, the three-dimensional topography clearly shows that the scanner is convex in the middle. Figure 5c indicates that the frame is about 30 μm higher than the substrate, but the mirror is almost flat, with its PV (peak to valley) being smaller than several hundred nanometers in the entire 4 mm diameter.

### 4.2. Fast Axis and Slow Axis Testing

**Figure 6:**
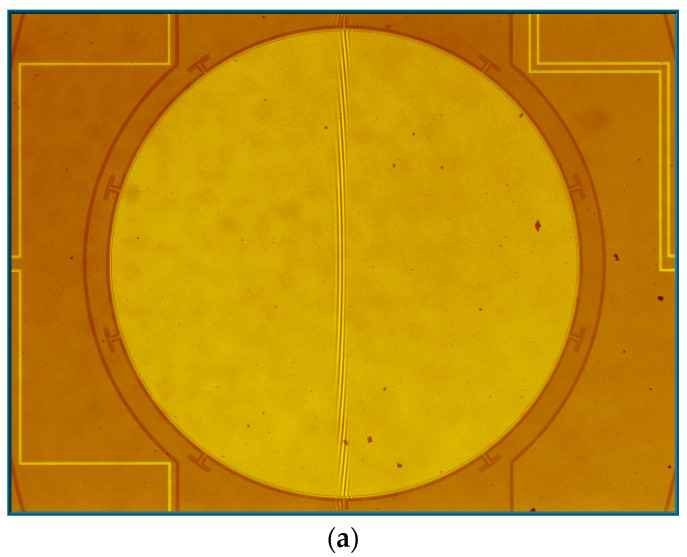

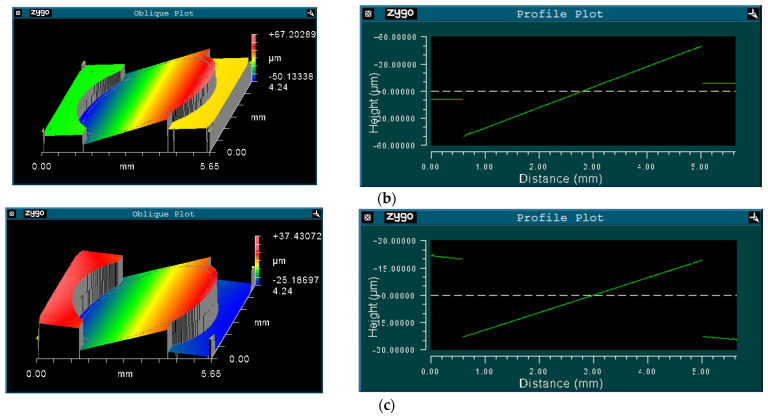
(**a**) Measurement area; (**b**) in-phase mode; and (**c**) out-of-phase mode.

The Dynamic Metrology Module (DMM) of the Zygo profiler was adopted to measure the mechanical scanning angle at each vibration frequency. DMM can freeze-frame the high-frequency vibration of the prototype by synchronizing a strobe light source in the profiler to the scanner movement. Figure 6a displays the micrograph of the measurement area and Figure 6b,c give the measured freeze-framed IP and OP modes of the fast axis of the scanner. The cross-sectional views clearly display the relative position of the mirror and the frame.

**Figure 7:**
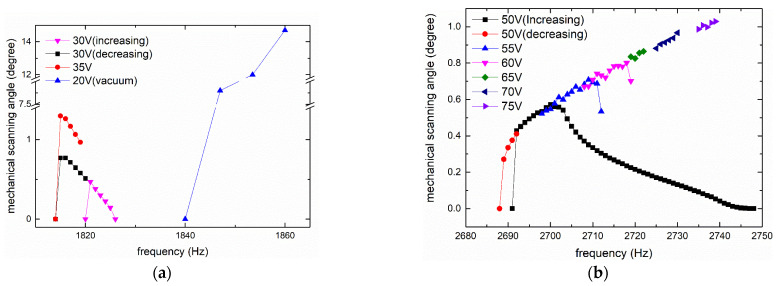
Measurement results of fast axis: (**a**) IP; and (**b**) OP.

The measurement results of fast axis mechanical scanning angle under different frequencies and voltages are illustrated in Figure 7. As a result of parametric resonance [27,28], the frequency of the excitation voltage was twice the resonant frequency of the scanner, and the horizontal axis in Figure 7 was one-half the excitation frequency; that is, the oscillation frequency of the measured prototype.

The results of IP mode are shown in Figure 7a. As the two curves under the 0–30 V square wave show, the result was a typical hysteretic frequency response (i.e., the curves for increasing frequency and decreasing frequency did not overlap, and the latter was wider). The peak of the curves was in the non-overlapping area. From the 35 V curve for decreasing frequency, the resonant frequency of IP mode was 1815 Hz and the mechanical scanning angle was ±1.3°.

Figure 7b illustrates the data for OP mode. As the two curves under 50 V voltage show, the response was also hysteretic and the curve for decreasing frequency was also wider; however, unlike IP mode, the data in the non-overlapping area were smaller than the data in the overlapping area, such that the peak was in the overlapping area. This provides an advantage. In contrast, the scanning mirror working in the non-overlapping area is very unstable and sensitive to the initial conditions, such that additional feedback control is needed.

Only part of the data that contained the resonant peak was recorded and plotted in the five curves (from 55–75 V) for increasing frequency. The resonant frequency of OP mode was about 2739 Hz and a mechanical scanning angle of ±1.03° was achieved under a 0–75 V square wave. The test frequencies of IP and OP modes were slightly lower than the theoretical and simulation results, mainly because the actual device had residual stress and metal layers, which were not considered in the theory and simulation. Moreover, the angles of the two modes were different, where the angle of the IP mode was larger under the same voltage.

An interesting phenomenon in the amplitude–frequency curve of the OP mode is that it did not display the typical curve, where the amplitude first rises sharply and then decreases slowly with the increase in frequency, similar to the curve in IP mode and throughout the literatures. In the narrow frequency band near the peak, its shape is reversed, rising slowly and falling sharply. To the best of our knowledge, the curve of this shape has not been reported in the literature. We guess that this phenomenon may be due to the large relative angle between the mirror and the frame in out-of-phase mode, which leads to a non-linear spring effect. As expressed in [29], negative and positive non-linear parameters will result in two opposite curves. For a system coupling mechanical domain and electrostatic domain, [30] proved that the sign of non-linear parameters can be changed between positive and negative under different driving conditions.

**Figure 8:**
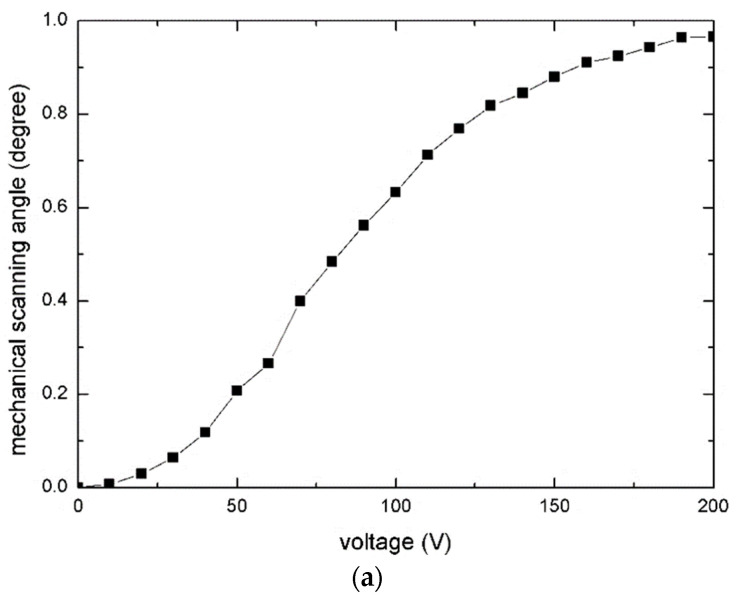

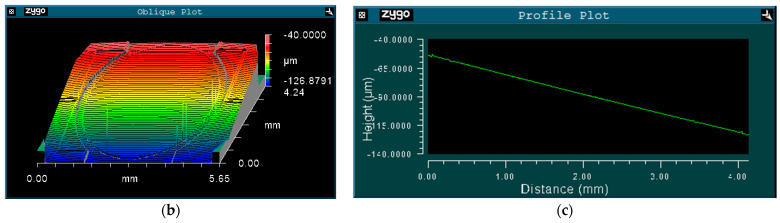
Measurement result for the slow axis: (**a**) Angle–voltage curve; (**b**) The torsion around the slow axis; and (**c**) Cross-sectional view of (**b**) along the fast axis.

Figure 8a depicts the quasistatic angle–voltage characteristic of the slow axis. The maximum mechanical scanning angle was ±0.97° under 200 V DC (Figure 8b,c).

### 4.3. 2D Scanning and Vacuum Testing

**Figure 9:**
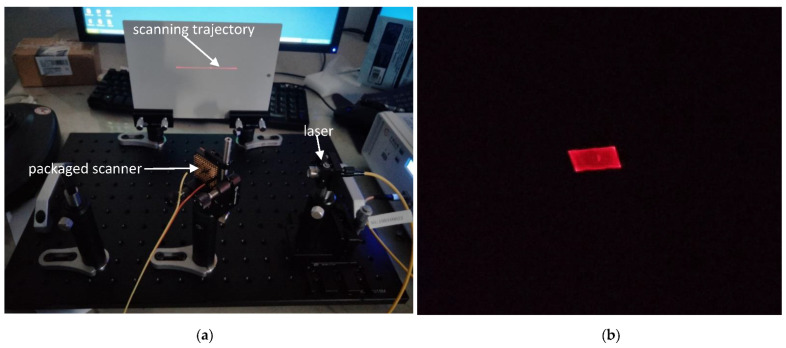
Two-dimensional beam scanning: (**a**) Experimental setup; and (**b**) Raster pattern.

In this section, 2D beam scanning based on the developed sample is demonstrated. Figure 9a shows the setup of the laser scanning system and Figure 9b shows the 2D raster scanning pattern.

**Figure 10:**
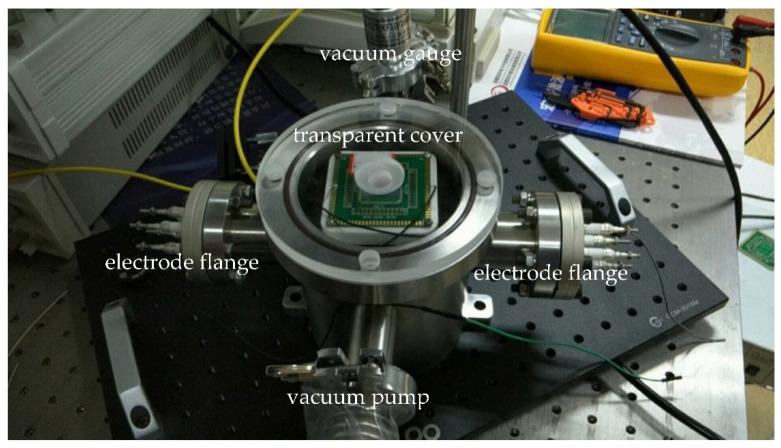
Vacuum measuring system.

The resonance of the fast axis was very sensitive to air damping. For measuring the scanner response in vacuum, a vacuum chamber was designed and manufactured, as shown in Figure 10. It was a stainless-steel cylinder with a transparent fused silica cover. There were four symmetrical channels on the side; two channels connected to the vacuum pump and vacuum gauge, while the other two channels were only open to the electronic driving signal.

**Figure 11:**
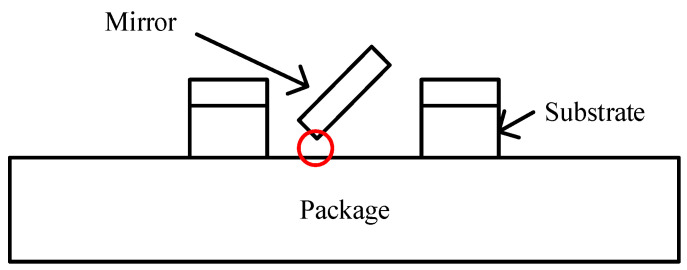
Contact between the mirror and the package.

The Zygo profiler could not measure the scanning angle through the transparent cover and, so, the scanning spot width was measured to calculate the angle. Under 20 V voltage and 1 Pa pressure, the scanning mirror started to scan as the frequency increased to 1840 Hz. When the frequency reached 1860 Hz, the scanning angle reached ±14.7°. The frequency continued to increase to 1862 Hz, when the device broke. Unlike ordinary damage (where an individual comb finger or beam is broken), this time, the entire mirror fell off. We guess that the angle exceeded 14.7° at 1862 Hz and the mirror touched the package during high-speed vibration, as shown in Figure 11. The aperture of the mirror was 4 mm and, so, the downward displacement of the scanner edge is 2sin(14.7°) ≈ 500 μm. Considering that the thickness of mirror and substrate were 75 μm and 400 μm, respectively, and the lift height was 30 μm, this reasoning is reliable.

In vacuum, the angle increased one order of magnitude with lower voltage. The measurement data are also plotted in Figure 7a. Thanks to the removal of air damping, the resonant frequency of IP mode increased a little, from 1815 Hz to 1860 Hz (or even greater).

## 5. Discussion

If a larger angle of slow axis is expected, the height difference between the moving comb and fixed comb of the vertical comb-drive actuator needs to be increased, which can be acquired through extending the length of the two arms or increasing the residual compressive stress. Extending the arms is a method in the design stage, which is simpler and more free than other process-related methods.

The lengths of the two arms influence the frequency of the frame around the fast axis. By tuning the frequency of the mirror around the fast axis at the same time, the change of the frequencies of IP and OP modes can be partly eliminated. Furthermore, in vacuum, the ratio of the angle of the mirror around the fast axis to the angle of the frame around the fast axis (viz. the mechanical-coupling gain *M*) is determined by the following formula [24]:

(6) $$M\left(\omega \right)=\left|\frac{\Theta_{i}\left(\omega \right)}{\Theta_{o}\left(\omega \right)}\right|=\frac{k_{i}}{\sqrt{{\left(k_{i}-J_{i}\omega^{2}\right)}^{2}}},$$

where *Θ_i_* and *Θ_o_* are the angle of the mirror and frame around the fast axis, respectively, and *ω* is the vibration angular frequency. As the formula expresses, the closer *ω_IP_* and *ω_OP_* are to *ω_i_*, the greater the gain. As a result, for the operating frequency specified by a development target, the angle can be optimized by adjusting the structural parameters related to the spring constant and mass moment of inertia.

In summary, the design, simulation, fabrication, and testing of a 2D scanning micromirror were presented in this paper. The resonant fast axis has two vibration modes, in-phase and out-of-phase, with frequencies of 1860 Hz and 2739 Hz, respectively. In the vacuum environment, a maximum total optical scan angle of 58.8° was reached. The non-resonant slow axis employed vertical comb-drive actuators, formed by the residual stress of the silicon dioxide membrane. The TOSA of the slow axis was about 4°.

## Figures and Tables

**Table 1:** Resonant frequencies.

Method	IP Mode (Hz)	OP Mode (Hz)
Calculation	2180	3391
Simulation	1930	2992

**Table 2:** Parameters of the fabrication process.

Layer	Thickness (μm)	Stress (MPa)
Substrate	400	
Buried oxide	1	
Silicon	75	
Deposited oxide	1	−400
Metal	0.05Cr/Ti + 0.5Au	

**Figure A1:**
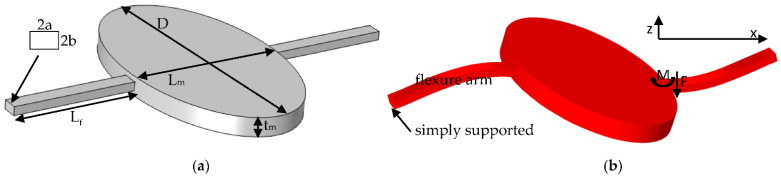
Schematic diagram of the torsion of the frame around the slow axis: (**a**) Structural parameters; and (**b**) Rocking mode.

## Data Availability

The data that support the findings of this study are available from the corresponding author upon reasonable request.

## Appendix A

The torsion of the frame around fast axis was called the out-of-plane rocking mode in [25], as illustrated in Figure A1. The definitions of all structural parameters (Figure A1a) are the same as those in [25]. The only difference is that the end of the arm is clamped in the reference, while being simply supported in this work, due to its connection to a softer beam. Consequently, the spring constant *k_o_* and mass moment of inertia *J_o_* of this mode are derived again in the following.

As the inset in Figure A1b shows, a co-ordinate system is constructed along the right arm. The differential equation of its deflection is [31]

(A1) w’’’’(x)=0.

The right end of the arm is simply supported and the boundary conditions are

(A2) $$w\left(L_{f}\right)=0,$$

(A3) $$w’’\left(L_{f}\right)=0.$$

The deflection, slope, moment, and force of the left end of the arm are

(A4) $$w\left(0\right)=-\frac{L_{m}\theta_{m}}{2},$$

(A5) $$w’\left(0\right)=-\theta_{m},$$

(A6) $$w’’\left(0\right)=\frac{M}{EI},$$

(A7) $$w’’’\left(0\right)=-\frac{F}{EI},$$

where *θ_m_* is the scanning angle of the frame; *M* and *F* are the moment and force exerted by the frame on the left end of the arm, respectively; *E* is the Young’s Modulus of the arm; and

(A8) $$I=\frac{\left(2a\right){\left(2b\right)}^{3}}{12}=\frac{4ab^{3}}{3}.$$

According to the equation and boundary conditions, the deflection and the interactions can be solved as

(A9) $$w\left(x\right)=\frac{3\theta_{m}}{L_{f}^{3}}\left(\frac{L_{m}}{2}+L_{f}\right)\left(-\frac{1}{6}x^{3}+\frac{L_{f}}{2}x^{2}\right)-\theta_{m}x-\frac{L_{m}\theta_{m}}{2},$$

(A10) $$M=FL_{f},$$

(A11) $$F=\frac{3\theta_{m}EI}{L_{f}^{3}}\left(\frac{L_{m}}{2}+L_{f}\right).$$

The reaction force on the frame is –*F* and –*M*. Combining two arms, the torque on the frame is

(A12) $$T=-2\left(M+\frac{FL_{m}}{2}\right).$$

Finally, the spring constant is

(A13) $$k_{o}=\frac{-T}{\theta_{m}}=\frac{2}{\theta_{m}}\left(M+\frac{FL_{m}}{2}\right)=\frac{2F}{\theta_{m}}\left(L_{f}+\frac{L_{m}}{2}\right)=\frac{6EI}{L_{f}^{3}}{\left(\frac{L_{m}}{2}+L_{f}\right)}^{2}.$$

Next, we solve the mass moment of inertia. First, the velocity is

(A14) $$\frac{dw\left(x\right)}{dt}=\frac{d\theta_{m}}{dt}\left[\frac{3}{L_{f}^{3}}\left(\frac{L_{m}}{2}+L_{f}\right)\left(-\frac{1}{6}x^{3}+\frac{L_{f}}{2}x^{2}\right)-x-\frac{L_{m}}{2}\right].$$

The kinetic energy of the right arm is

(A15) $$KE_{f}=\int_{0}^{L_{f}}\frac{1}{2}{\left(\frac{dw\left(x\right)}{dt}\right)}^{2}dm=\int_{0}^{L_{f}}\frac{1}{2}{\left(\frac{dw\left(x\right)}{dt}\right)}^{2}\rho \left(2a\right)\left(2b\right)dx,$$

where *ρ* is the density of the arm. Substituting expression (A14) into (A15) results in

(A16) $$KE_{f}={\left(\frac{d\theta_{m}}{dt}\right)}^{2}\frac{M_{f}}{2}\left(\frac{2}{105}L_{f}^{2}+\frac{3}{35}L_{f}L_{m}+\frac{17}{140}L_{m}^{2}\right)={\left(\frac{d\theta_{m}}{dt}\right)}^{2}\frac{J_{f}}{2},$$

where

(A17) $$M_{f}=4\rho abL_{f}.$$

Thus, the mass moment of inertia is

(A18) $$J_{o}=J_{m,yy}+2J_{f},$$

where *J_m,yy_* is the mass moment of inertia of the frame. For an elliptical frame, this can be expressed as

(A19) $$J_{m,yy}=\frac{M_{m}}{12}\left(\frac{3}{4}L_{m}^{2}+t_{m}^{2}\right),$$

where *M_m_* is the mass of the frame, which can be expressed as

(A20) $$M_{m}=\frac{\pi }{4}\rho L_{m}Dt_{m}.$$
